# Integrative analysis of the pharmaceutical active ingredient and transcriptome of the aerial parts of *Glycyrrhiza uralensis* under salt stress reveals liquiritin accumulation via ABA-mediated signaling

**DOI:** 10.1007/s00438-021-01847-1

**Published:** 2022-02-20

**Authors:** Quan Bi, Hua Yao, Fei Wang, Dajun He, Wenbin Xu, Shuangquan Xie, Xifeng Chen, Yuxia Li, Hailiang Liu, Haitao Shen, Hongbin Li

**Affiliations:** 1 Key Laboratory of Xinjiang Phytomedicine Resource and Utilization of Ministry of Education, College of Life Sciences, Shihezi University, Shihezi, 832003 China; 2 Key Laboratory of Oasis Town and Mountain-Basin System Ecology of Xinjiang Production and Construction Corps, Shihezi University, Shihezi, 832003 China; 3 Institute of Materia Medica of Xinjiang, Urumqi, 830004 China; 4 Institute for Regenerative Medicine, Shanghai East Hospital, Tongji University School of Medicine, Shanghai, 200123 China

**Keywords:** *Glycyrrhiza uralensis* aerial parts, Salt stress, Transcriptome analysis, Liquiritin biosynthesis, ABA signaling

## Abstract

The aerial parts of *Glycyrrhiza uralensis* supply substantial raw material for the extraction of active pharmaceutical ingredients comprehensively utilized in many industries. Our previous study indicated that salt stress increased the content of active ingredients. However, the regulatory mechanism remains unclear. In this study, RNA-sequencing (RNA-seq) of the aerial parts of *G. uralensis* treated with 150 mM NaCl for 0, 2, 6, and 12 h was performed to identify the key genes and metabolic pathways regulating pharmacological active component accumulation. The main active component detection showed that liquiritin was the major ingredient and exhibited more than a ten-fold significant increase in the 6 h NaCl treatment. Temporal expression analysis of the obtained 4245 differentially expressed genes (DEGs) obtained by RNA-seq revealed two screened profiles that included the significant up-regulated DEGs (UDEGs) at different treatment points. Kyoto Encyclopedia of Genes and Genomes (KEGG) analysis of these UDEGs identified phenylpropanoid metabolism and flavonoid biosynthesis as the most significantly enriched pathways in 2 h treated materials. Interestingly, the carotenoid biosynthesis pathway that is related to ABA synthesis was also discovered, and the ABA content was significantly promoted after 6 h NaCl treatment. Following ABA stimulation, the content of liquiritin demonstrated a significant and immediate increase after 2 h treatment, with the corresponding consistent expression of genes involved in the pathways of ABA signal transduction and flavonoid biosynthesis, but not in the pathway of glycyrrhizic acid biosynthesis. Our study concludes that salt stress might promote liquiritin accumulation through the ABA-mediated signaling pathway, and provides effective reference for genetic improvement and comprehensive utilization of *G. uralensis*.

**Supplementary Information:**

The online version contains supplementary material available at 10.1007/s00438-021-01847-1.

## Introduction

As the most widely used Chinese traditional medicinal plant (Kitagawa 2002), licorice has comprehensive pharmacological activities for disease therapy including anti-tumor, anti-virus, and memory enhancement performed by its active pharmaceutical components (Shim et al. 2000; Dhingra et al. 2004; Dhingra and Sharma 2006; Kim et al. 2006; Lee et al. 2007; Nagai et al. 2007; Sato et al. 2007; Shin et al. 2007; Fiore et al. 2008; Tohma and Gulcin 2010; Jiang et al. 2018). Flavonoid compounds are considered to be the main active ingredients containing liquiritin that has recently been validated to play important roles in inhibiting the replication of novel coronavirus (Zhu et al. 2020). These new discoveries of pharmacological function will further enhance the importance of licorice. The demand for licorice in the international market will also be further increased. In recent years, with the severe decrease of wild licorice worldwide, cultivated licorice has become the main supply source of raw roots for utilization. However, a long growth cycle of three to four years of licorice greatly limits the production of roots. As a perennial plant, licorice provides massive aerial parts annually that also include sufficient active pharmaceutical ingredients that are comprehensively utilized in many industries.

Flavonoids such as liquiritin belong to diverse secondary metabolites that exhibit significant positive correlations between the accumulation and moderate environmental stimulation (Liu et al. 2004, 2006, 2013; Li et al. 2011; Sun et al. 2012). As the most comprehensive utilized licorice, *G. uralensis* provides the majority of the raw materials of both aerial parts and roots used for the extraction of active pharmaceutical components, and usually grows in salt, drought, and high or low temperature stress environments (Qiao et al. 2017). There are many factors including abiotic stress, rhizosphere microorganisms, and endophytes that affect the synthesis and accumulation of active pharmaceutical components of *G. uralensis* (Afreen et al. 2005; Hayashi and Sudo 2009; Dang et al. 2020). Moderate salt stress effectively improved the content of glycyrrhizic acid and total flavonoids in *G. uralensis* (Wan et al. 2011; Tong et al. 2019). As an important secondary metabolite in plants, flavonoids have been validated to exert the antioxidant activity to directly scavenge reactive oxygen species (ROS) under stress conditions (Yamasaki et al. 1995; Moore et al. 2005). The plant hormone abscisic acid (ABA) is the key regulator that plays prominent roles for plant growth and development and in response to environmental stresses (Seo and Koshiba 2002; Zhang et al. 2006; Yoshida et al. 2015; Luo et al. 2021). Many studies have suggested that exogenous ABA positively influences the phytochemical content in selected plants. Application of ABA increased the accumulation of phenolics in muscadine grapes and of total phenolics and total anthocyanins in red lettuce (Sandhu et al. 2011; Buran et al. 2012), which are two major members of flavonoids, indicating the significant role of ABA in triggering flavonoid biosynthesis in plants.

Currently, the regulatory mechanism of the synthesis of flavonoids in *G. uralensis* aerial parts controlled by salt stress remains unclear. In this work, RNA-sequencing (RNA-seq)-based comparative transcriptome analysis of the aerial parts of *G. uralensis* treated with NaCl was performed, combined with the detections of active pharmaceutical components by ultra-performance liquid chromatography/tandem mass spectrometry (UPLC-MS/MS), to identify the key genes and regulatory mechanism controlling pharmacological active component accumulation. The significant up-regulated differentially expressed genes (UDEGs) distributed in the pathways of phenylpropanoid metabolism and flavonoid biosynthesis were discovered. Furthermore, ABA synthesis and signal transduction were also identified as the most significantly enriched pathways, and ABA content was induced after salt stimulation. Moreover, exogenous ABA promoted the expressions of genes involved in flavonoid biosynthesis pathway and increased liquiritin production. Overall, it is concluded that salt stress might promote liquiritin accumulation through the ABA-mediated signaling pathway.

## Materials and methods

### Plant materials and salt treatment

The *G. uralensis* seeds were treated with 98% concentrated sulfuric acid for 50 min to break seed dormancy, with a subsequent rinse (three times) with sterilized distilled water and disinfection by 0.1% of HgCl for 10 min. The sterilized seeds were germinated on vermiculite in an automatic climate chamber (200 µmol mm^−2^ s^−1^ light intensity, 16 h light/8 h dark photoperiod, 50–55% relative humidity, and 28 °C/25 °C day/night culture temperature). The 60-day-old *G. uralensis* seedlings were cultured for 5 days through hydroponics and were then treated with 150 mM NaCl in culture medium or with 50 mg/L ABA spraying as the treatments. The aerial parts were collected after 0, 2, 6, and 12 h of continuous treatment. For the ABA treatment, exogenous ABA was sprayed onto the aerial parts of *G. uralensis* seedlings. All the samples were washed with sterile water and frozen in liquid nitrogen immediately after collection and stored at 80 °C for further use. Three independent biological replicates in each treatment were performed with 15 seedlings in each group.

### RNA-sequencing and data assembly

The RNA of fresh aerial parts was extracted using a Plant RNA Purification Kit (TIANGEN, Beijin, China) according to the manufacturer’s instructions; the RNA was then utilized as the template to synthesize cDNA for quantitative real-time PCR (qRT-PCR) detection. High-quality RNA samples were used to construct the sequencing library and validate the RNA-seq data by qRT-PCR. RNA purification, reverse transcription, library construction, and sequencing were performed at Majorbio Bio-pharm Biotechnology Co., Ltd. (Shanghai, China), using the Illumina HiSeq 4000 platform according to the manufacturer’s instructions (Illumina, San Diego, USA). The obtained raw paired end reads were trimmed and quality controlled by SeqPrep (https://github.com/jstjohn/SeqPrep) and Sickle (https://github.com/najoshi/sickle) with default parameters. Then, clean data from the samples were used for de novo assembly with Trinity (http://trinityrnaseq.sourceforge.net/). TopHat2 (http://ccb.jhu.edu/software/tophat/index.shtml) and HISAT2 (http://ccb.jhu.edu/software/hisat2/index.shtml) were used to compare the clean data with the *G. uralensis* genome (http://ngs-data-archive.psc.riken.jp/Gur-genome/index.Pl).

### Screening of differential gene expression and functional enrichment analysis

The transcripts per million (TPM) method was used to identify differentially expressed genes (DEGs) between the control group and treatment group. RSEM (http://deweylab.biostat.wisc.edu/rsem/) was utilized to quantify gene and isoform abundances. The R statistical software package EdgeR (http://www.bioconductor.org/packages/2.12/bioc/html/edgeR.html) was used for the analysis of differential expression. KEGG Orthology-Based Annotation System (KOBAS) (http://kobas.cbi.pku.edu.cn/home.do) was used forKyoto Encyclopedia of Genes and Genomes (KEGG) pathway analysis.

### Determination of endogenous phytohormones and active pharmaceutical ingredients

The materials of aerial parts of *G. uralensis* were washed three times with deionized water and were then quickly ground in liquid nitrogen, and 1 g powder samples were added to a centrifuge tube with the addition of 5 mL 100% methanol for subsequent extraction by ultrasonic methods at 1000 W for 60 min at 25 °C. The supernatants were collected and transferred to a new centrifuge tube for centrifugation at 12,000 rpm for 5 min at 4 °C. The mixtures were filtered through filter paper. The extraction procedure was replicated two times, and all the filtered extract solutions were collected and placed in a 10 mL centrifuge tube for determination of active pharmaceutical ingredients.

Accurately weighed gibberellin A3 (GA3), licochalcone A, glabridin, abscisic acid (ABA), brassinolide (BR), isoliquiritigenin, zeatin (ZT), jasmonic acid (JA), glycyrrhetnic acid, liquiritin, indole-3-acetic acid (IAA), and glycyrrhizic acid were modulated to 1.0 mg/mL stock solution, which were then diluted with methanol to final concentrations of 1, 5, 10, 25, 50, 75, and 100 ng/mL as a mixed standard solution to produce based on Acquity UPLC H-class ultra-performance liquid chromatography, using a Xevo TQS triple quadrupole tandem mass spectrometer (UPLC-MS) (Supplementary Table S1). The separations were performed using a Waters ACQUITY UPLC BEH C^18^ column (50 mm × 5 mm, 1.7 μm, Waters, USA). The chromatographic conditions were as follows: Acquity UPLC BEH C18 column temperature 30 °C, flow rate 0.3 mL/min, and injection volume 1.0 μL. The final optimized mobile phase included 0.1% formic acid water (A) and acetonitrile (B) solutions, with the following gradient elution procedure: 0–3.0 min, 20–98% B; 3.0–4.5 min, 98% B; 4.5–5.0 min, 98–20% B; 5.0–6.0 min, 20% B. The mass spectrometric analysis was performed in multi-reaction monitoring (MRM) mode, with the optimal conditions as follows: source temperature of 200 °C, capillary voltage of 2300 V, source offset of 50 V, desolvation temperature of 450 °C. The flow of desolvation gas and cone gas was 750 L/Hr and 150 L/Hr, respectively. Waters MassLynx software (Version 4.1) was used for data acquisition and processing. The details of the mass spectrometric parameters of each component are presented in Supplementary Table S2, with the total ion chromatogram (TIC) diagram of the detected substances in Supplementary Fig. S1 were indicated.

### Validation of RNA-seq data by qRT-PCR

To validate the identified DEGs based on RNA-seq data, eight DEGs that control terpenoid backbone biosynthesis, carotenoid biosynthesis, phenylpropanoid biosynthesis and hormone signaling were selected for qRT-PCR analysis with the specific primers (Supplementary Table S3). The LightCycler^®^ 480 Real-Time PCR System (Roche Diagnostics International, Rotkreuz, Switzerland) was utilized for qRT-PCR assays with *Lectin* (Gene ID: Glyur000100s00008376) as the internal control for normalization. The relative expression values were determined by the 2^−ΔΔCt^ method, and the heatmap was generated by MultiExperiment Viewer software.

### Statistical analysis

The data of phytohormone and active pharmaceutical ingredient contents were analyzed by SPSS software (Version 13.0). The statistical analysis was performed by one-way analysis of variance (ANOVA) with Duncan’s test.

## Results

### Determination of main active components in aerial parts of *G. uralensis* under NaCl stress

**Fig. 1:**
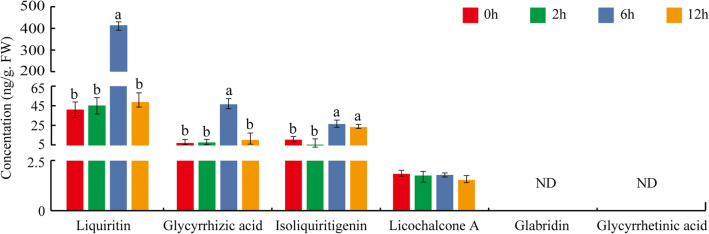
Determination of the content of the six main active components in NaCl-treated aerial parts of *G. uralensis*. The aerial parts of sixty-day-old *G. uralensis* plants treated with 150 mM NaCl for 0 h, 2 h, 6 h, and 12 h were collected for determination of the six main active components as presented using UPLC-MS/MS method. Different letters followed by mean ± standard error indicate significant differences at *p* < 0.05 level. *ND* No detection

Regarding the positive promotion of secondary metabolites in plants by salt stress (Bibi et al. 2019), we determined the six main active components in NaCl-treated aerial parts of *G. uralensis* including glycyrrhizic acid, glycyrrhetinic acid, liquiritin, licochalcone A, isoliquiritigenin, and glabridin. The results showed that, liquiritin, glycyrrhizic acid, and isoliquiritigenin were significantly induced after 6 h NaCl treatment, especially the liquiritin content, which exhibited more than ten-fold accumulation, without a change in the licochalcone A content (Fig. 1). Glycyrrhetinic acid and glabridin were not detected as the contents were lower than the limit of quantification in both normal and treated materials. These results suggest that liquiritin was the dominant active ingredient and the most preferential increased metabolite by NaCl stimulation.

### Identification and classification of differentially expressed genes in different treatment time of salt stress

To identify genes and metabolic pathways regulating the active component accumulation under salt stress, the aerial parts of *G. uralensis* treated with 150 mM NaCl for 0, 2, 6, and 12 h were used for high throughput sequencing analysis. Based on a comparison of the data of high-quality sequences and mapping to the reference genome of *G. uralensis*, more than 83.28% of clean reads of the transcriptome data in all samples were uniquely mapped to the genome of *G. uralensis* (Supplementary Table S4). qRT-PCR validation of seven genes involved in terpenoid backbone biosynthesis, carotenoid biosynthesis, phenylpropanoid biosynthesis, and plant hormone signaling transduction indicated their consistent expression profiles with the RNA-seq data, and combined with the correlation analysis of qRT-PCR and RNA-seq, showed the high reliability of the transcriptome data (Supplementary Fig. S2).

**Fig. 2:**
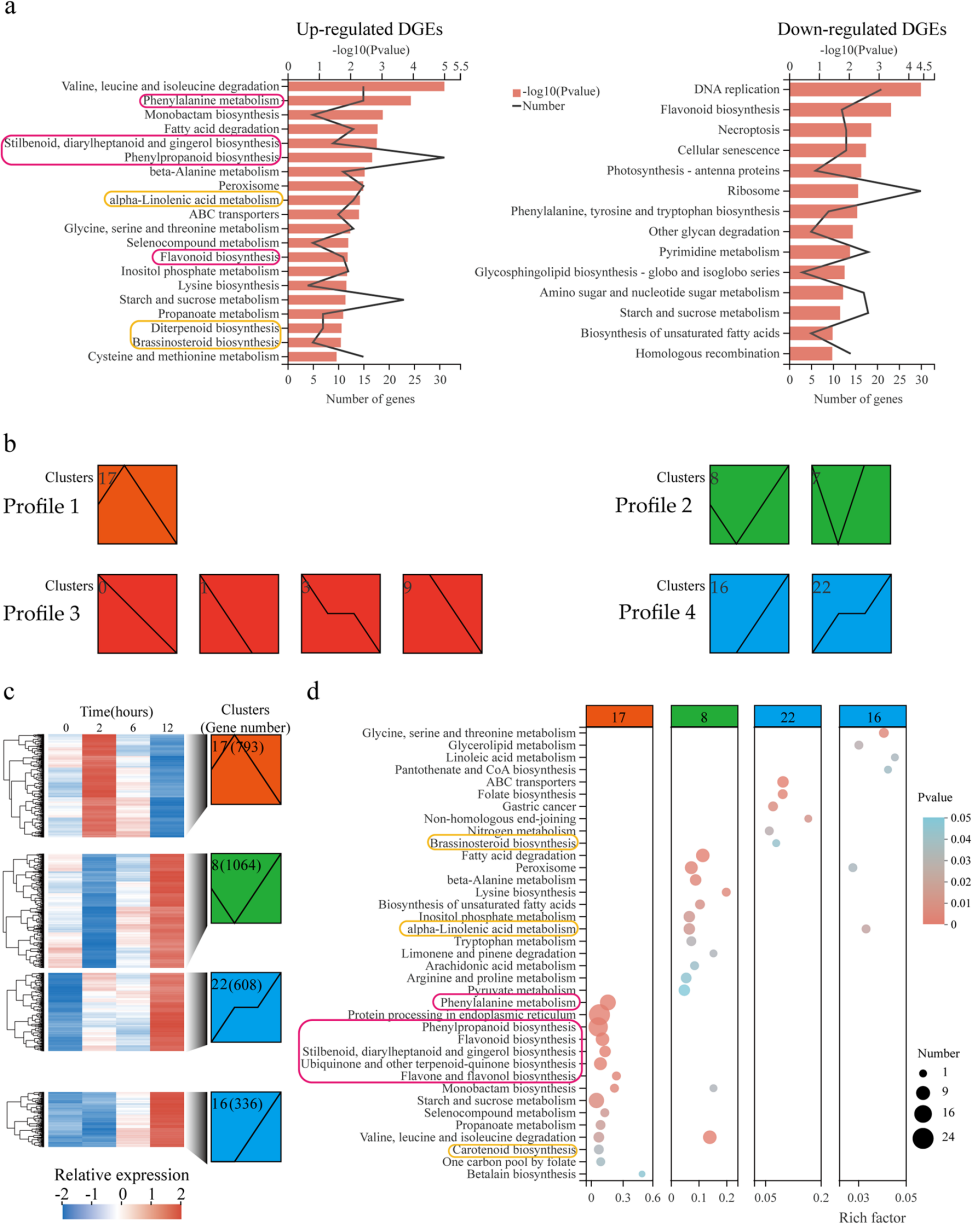
Transcriptome analysis of aerial parts of *G. uralensis* under salt stress at different times. The aerial parts of sixty-day-old *G. uralensis* plants treated with 150 mM NaCl for 0, 2, 6, and 12 h were collected for generation of RNA-sequencing (RNA-seq) data, and the identified DEGs were used for further analysis. **a** KEGG enrichment of up- and down-regulated DGEs. Purple and yellow line boxes represent the pathways of flavonoid biosynthesis and phytohormone synthesis, respectively. **b** Temporal expression pattern analysis of the DEGs. The Short Time-Series Expression Miner (STEM) program classified the 4525 DEGs into four main possible model profiles according to the temporal gene expression patterns, with orange, green, red, and blue colors to denote different profiles. **c** Clustering heatmap of DGEs in different clusters. Clusters 8, 16, 17, and 22 that showed up-regulated DEGs at different treatment time points of NaCl stress were selected for analysis. **d** KEGG enrichment pathway analysis of the DEGs involved in clusters 8, 16, 17, and 22. Purple and yellow line boxes denote the pathways of flavonoid biosynthesis and phytohormone biosynthesis, respectively

Through the filter condition of |Log_2_FC|> 2 (*p* < 0.05) between the treatment group and the control group of the RNA-seq data, a total of 4245 differentially expressed genes were obtained (Supplementary Fig. S3). KEGG enrichment analysis of the up-regulated DGEs (UDEGs) and down-regulated DGEs showed that, the related pathways of flavonoid synthesis and endogenous hormone synthesis were mainly significantly enriched in the UDGEs (Fig. 2a). To further analyze the possible role of these DEGs, temporal expression analysis was performed using the Short Time-Series Expression Miner (STEM) program under the condition of *p* < 0.05, generating four main possible model profiles including nine significantly different gene expression patterns to be recognized (Fig. 2b). There were 2072, 2642, 793, and 944 DEGs distributed in the four profiles marked by red, green, orange and blue colors respectively. DEGs of clusters 8, 16, 17, and 22 showed significant up-regulation at different treatment times of NaCl stimulation (Fig. 2b). KEGG enrichment analysis of the DGEs in the four clusters indicated that pathways of secondary metabolic substance biosynthesis and metabolism including phenylalanine metabolism (thirteen genes), phenylpropanoid biosynhtesis (twenty genes), flavonoid biosynthesis (nine genes), stilbenoid, diarylheptanoid and gingerol biosynthesis (six genes), flavone and flavonol biosynthesis (three genes), etc., were identified as the significantly enriched pathways in cluster 17 (Fig. 2 c,d, Supplementary Table S5), showing the reasonable close connection between the expressions of these genes at 2-h time point and the accumulation of the active components at 6-h time point. Remarkably, four genes involved in the pathway of carotenoid biosynthesis that is related to ABA synthesis were also significantly enriched, implying the potential existing regulation link between ABA signaling and active ingredient production.

### Analysis of liquiritin biosynthesis under salt stress

**Fig. 3:**
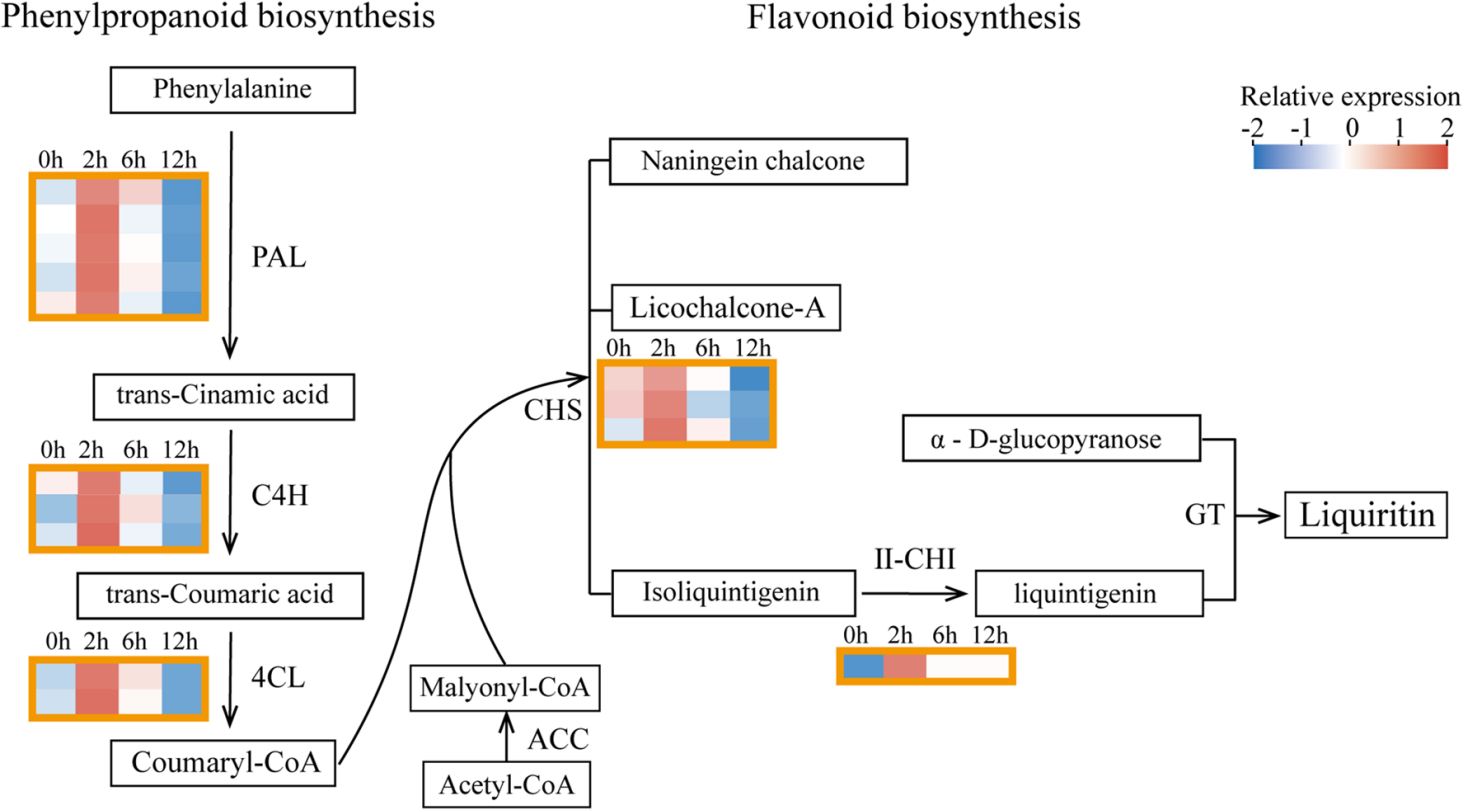
Expression analysis of the DGEs involved in the liquiritin synthesis pathway. The genes involved in liquiritin synthesis were selected from the reported studies (Koes et al. 1994; Shimada et al. 2003; Bibi et al. 2019). The expression profiles of the detectable DEGs were presented, with pink and blue colors to denote the high or low expression levels. *PAL* phenylalanineammonialyase, *C4H* Trans-cinnamate 4-monooxygenase, *4CL* 4-coumarate-CoA ligase, *ACC* Acetyl-CoA carboxylase, *CHS* Chalcone synthase, II-CHI: type II Chalcone–flavonone isomerase, *GT* glycosyltransferase

To investigate the effect of salt stress on the expressions of genes involved in liquiritin biosynthesis, we performed the expression analysis of the relevant genes locating in the liquiritin biosynthesis pathway enriched in each model profile. Further analysis of genes with more than 10 TPM expressions was conducted (Supplementary Table S6). In the process from phenylalanine to flavonoid, a total of 14 DEGs, i.e., five *PALs*, three *4CLs*, two *C4Hs*, three *CHSs*, and one II*-CHI*, were discovered in the significantly enriched pathways of cluster 17 and displayed increased expressions at 2-h point of treatment time with NaCl stress (Fig. 3). These results suggest that these genes might play important role in controlling liquiritin biosynthesis under salt stress.

As a result of the significant increase of glycyrrhizic acid content under NaCl stress (Fig. 1), we performed the expression analysis of the DEGs locating in the glycyrrhizic acid biosynthesis pathway enriched in each model profile. Of the seven genes involved in glycyrrhizic acid biosynthesis, only three genes including *SQLE* (Gene ID: Glyur000270s00013301) and *SQS2* (Gene ID: Glyur000017s00002413) that showed significant accumulation at 2-h treatment time point and *CYP72A154* (Gene ID: Glyur000758s00019978) that indicated significantly up-regulated expression at the 12-h treatment time point were detected (Supplementary Fig. S4, Supplementary Table S7). The other genes that distribute in the glycyrrhizic acid biosynthesis pathway catalyzing the synthesis of intermediates and the final production of glycyrrhizic acid were expressed extremely low levels or were not detectable (Supplementary Fig. S4, Supplementary Table S7), suggesting a complex regulatory mechanism of glycyrrhizic acid biosynthesis in the aerial parts of *G. uralensis*.

### Analysis of ABA biosynthesis and signal transduction under salt stress

**Fig. 4:**
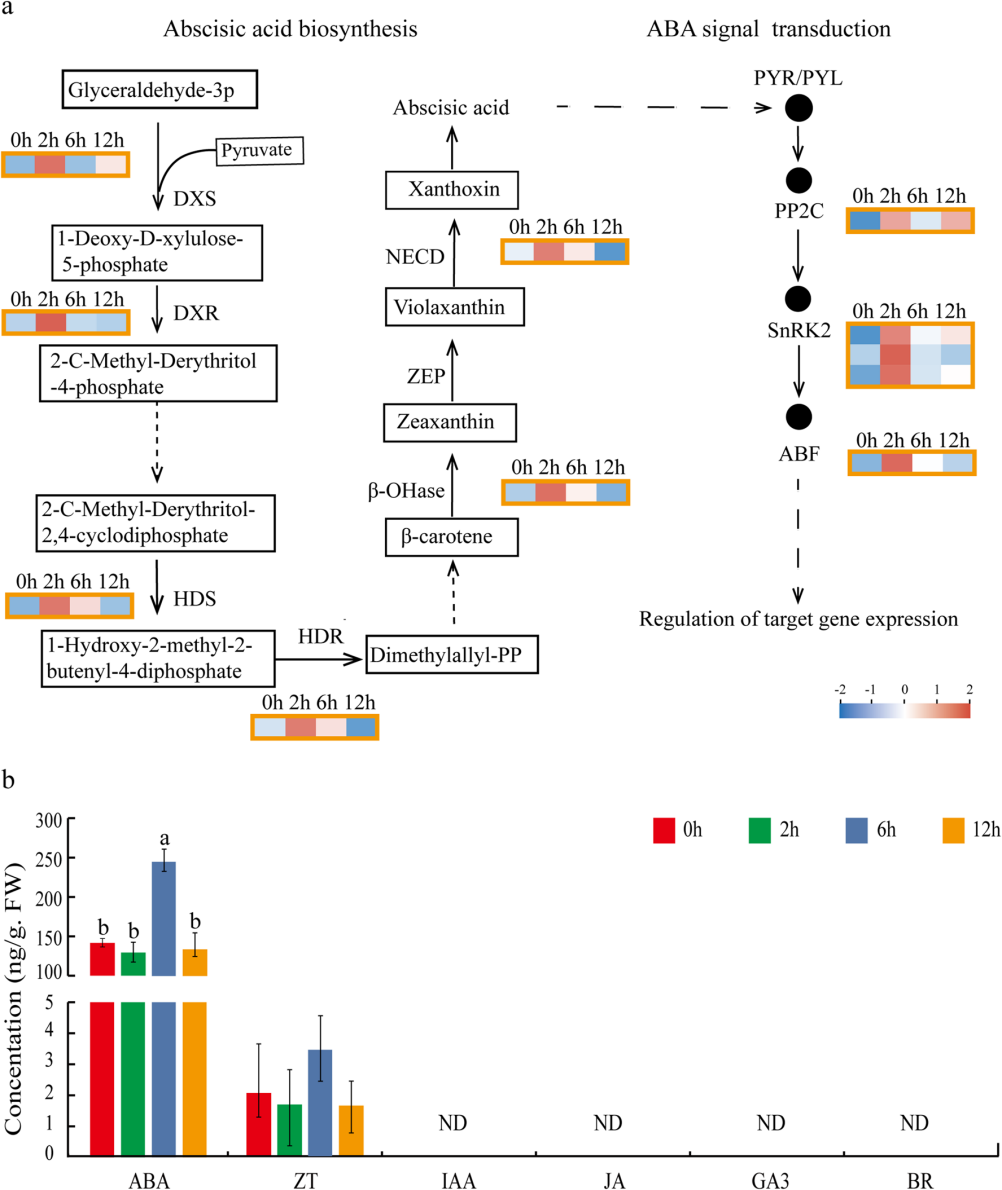
Expression analysis of the DGEs involved in ABA biosynthesis and signal transduction and detection of six main phytohormones under salt stress. The genes that participated in ABA biosynthesis and signal transduction were selected for further analysis (Yamasaki et al. 1995; Ma et al. 2009; Park et al. 2009; Umezawa et al. 2010). **a** Expression analysis of the DGEs involved in ABA biosynthesis and signal transduction pathways. The expression profiles of the detectable DEGs were indicated, and the pink and blue colors denote high or low expression levels. **b** Determination of the six main endogenous phytohormones. Different letters represent significant differences at the *p* < 0.05 level. *ND* Not detected, *DXS* 1-deoxy-D-xylulose 5-phosphate synthase, *DXR* 1-deoxy-D-xylulose 5-phosphate reductoisomerase, *HDS* 4-hydroxy-3-methylbut-2-en-1-y-l-diphosphate synthase, *HDR* 1-deoxy-D-xylulose-5-phosphate reductoisomerase, *β-OHase* Beta-carotene hydroxylase, *ZEP* Carotenoid biosynthesis, *NCED* Carotenoid cleavage dioxygenase, *PP2C* Protein phosphatase 2C, *SnRK2* Serine/threonine-protein kinase, *ABF* ABRE binding factor

Regarding the abscisic acid (ABA) biosynthesis pathway as one of the most significantly enriched pathways (Fig. 2b, d) and the important role of ABA in plants in response to abiotic stress (Lata and Prasad 2011; Yoshida et al. 2015), to investigate the possible function of ABA in regulating active ingredient production, we analyzed the expressions of genes participating in ABA biosynthesis and signal transduction. A total of six genes were enriched in the ABA synthesis pathway: 1-deoxy-D-xylulose (DXS), 1-deoxy-D-xylulose 5-phosphate reductoisomerase (DXR), 4-hydroxy-3-methylbut-2-en-1-y-l-diphosphate synthase (HDS), 1-deoxy-D-xylulose-5-phosphate reductoisomerase (HDR), Beta-carotene hydroxylase (β-OHase), and carotenoid cleavage dioxygenase 1 (NCED1), and appeared a significantly induced accumulation in 2 h treated materials, suggesting their potential important effect on ABA biosynthesis and the salt stress response (Fig. 4a, Supplementary Table S8). Moreover, three genes located in ABA signal transduction pathway, *PP2C*, *SnRK2*, and *ABF*, also had significantly elevated expressions in 2-h treated materials. These results indicate that ABA biosynthesis and signal transduction were the most important phytohormone signaling components in the process of *G. uralensis* in response to salt stimulation. The pathways of diterpenoid biosynthesis, brassinosteroid biosynthesis, carotenoid biosynthesis, and alpha-Linolenic acid metabolism were enriched in up-regulated clusters, providing the potential role of UDEGs of these clusters to involve in the biosynthesis of GA, BR, ABA, and JA (Fig. 2b). Thus, the contents of the main endogenous phytohormones under salt stress including ABA, GA3, JA, ZT, IAA, and BR were measured, showing the interesting result that, ABA was the only major detected hormone and displayed a dominant increase in 6 h treated materials (Fig. 4b), with the other phytohormones of ZT being present at a low content and of GA3, JA, IAA, and BR being undetectable.

### Effect analysis of ABA on the content of active ingredients in *G. uralensis*

**Fig. 5:**
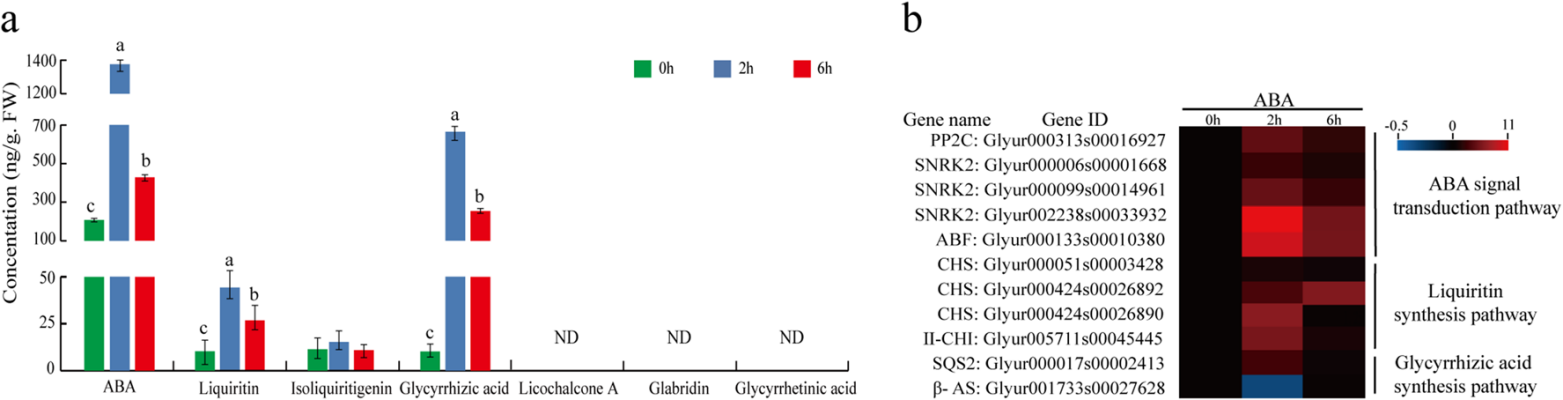
Analyses of active ingredient contents and related gene expressions under exogenous ABA treatment. **a** Determination of intrinsic ABA and six main active ingredients in the aerial parts of *G. uralensis* treated with exogenous ABA application. Different letters indicate significant differences at the *p* < 0.05 level. *ND* Not detected. **b** qRT-PCR-based heatmap of expressions of the genes related to the signaling pathways of ABA signal transduction, flavonoid synthesis, and glycyrrhizic acid synthesis. The genes that were detectable in the RNA-seq data were selected for qRT-PCR assays

To further understand the effect of ABA on the content of active components in *G. uralensis*, we determined the content of six main active ingredients in the aerial parts of *G. uralensis* treated by exogenous ABA for 0 h, 2 h, and 6 h. The results indicated that, after exogenous ABA application, the endogenous ABA content showed more than a seven-fold increase after 2 h treatment, displaying the high effectiveness of the treatment. The contents of both liquiritin and glycyrrhizic acid were promoted significantly, with no change in the content of isoliquiritigenin and no detection of glycyrrhetinic acid, licochalcone A, and glabridin (Fig. 5a). On the basis of the RNA-seq data (Supplementary Table S9), qRT-PCR-based expression analysis of the key genes distributing in the pathways of ABA signal transduction, liquiritin biosynthesis, and glycyrrhizic acid biosynthesis indicated that, of all the detectable genes, *PP2C*, *SnRK2*, and *ABF* locating in ABA signal transduction, and PAL, CHS, and CHI involved in liquiritin synthesis appeared immediate induced accumulation after 2 h ABA treatment, while *SQS2* and *β-AS* in the pathway of glycyrrhizic acid synthesis presented relatively low normal or down-regulated expression levels respectively (Fig. 5b). These results suggest that ABA could stimulate the accumulation of liquiritin by inducing the expressions of genes involved in the pathways of ABA signal transduction and flavonoid synthesis.

## Discussion

Environmental stress acts as a positive regulator to stimulate the production of pharmacological active components that are the important members of secondary metabolites in medicinal plants (Li et al. 2012). *G. uralensis* plants usually grow under stress conditions of arid and semiarid areas, and salt stress significantly increases the accumulation of secondary metabolites of glycyrrhizic acid and flavonoids (Liu et al. 2013; Qiao et al. 2017). Low-concentration NaCl increased the accumulation of pharmacological active components, especially the significant enrichment of flavonoid by promoting β-AS and CHS expression (Wang et al. 2019). In this study, of the six main active ingredients in *G. uralensis*, the contents of liquiritin, glycyrrhizic acid, and isoliquiritigenin in the aerial parts of *G. uralensis* were significantly enriched under 6-h and 12-h NaCl stimulation (Fig. 1). The biosynthesis and production of liquiritin and glycyrrhizic acid were regulated by relevant genes in the corresponding pathways (Chandran et al. 2019; Ma et al. 2019). By RNA-seq-based comparative transcriptome analysis, a total of 4245 DEGs were identified in the aerial parts of *G. uralensis* under salt stress, and were subjected to KEGG analysis, which indicated phenylalanine metabolism, phenylpropanoid biosynthesis, flavonoid biosynthesis, flavone and flavonol biosynthesis as the significantly enriched pathways (Figs. 1, 2), implying these pathways and DEGs play important roles in liquiritin accumulation. It has been reported that salt stress stimulates the expression of genes related to flavonoid synthesis and increases the content of flavonoids (Zorb et al. 2013; Ben Abdallah et al. 2016; Mortimer et al. 2016; Ma et al. 2019; Wang et al. 2019). In this study, similar results were obtained that the related genes locating in the pathways of phenylpropanoid biosynthesis and flavonoid biosynthesis including *PAL*, *C4H*, *4CL*, *CHS*, and II*-CHI* were significantly increased in 2 h treated materials by NaCl (Fig. 3).

ABA is the major stress-responsive phyto-hormone and plays important roles in regulating the synthesis of cellular secondary metabolites in response to abiotic stress (Zorb et al. 2013; Ben Abdallah et al. 2016; Ma et al. 2019). The accumulation of ABA under salt stress is a cell signaling process, encompassing initial stress signal perception, cellular signal transduction, and regulation of expression of genes encoding key enzymes in ABA biosynthesis and metabolism. The carotenoid biosynthesis pathway that directly results in ABA biosynthesis was identified as one of the most significantly enriched pathways in NaCl-treated materials (Fig. 2b). Carotenoids are precursors of ABA synthesis, and *β-OHase* and *NCED* are two key genes that catalyze carotenoids as a precursor to generate ABA (Schwartz et al. 1997; Iuchi et al. 2000; Mortimer et al. 2016). In this study, two DEGs of *β-OHase* and *NECD* involved in the significantly enriched pathway of carotenoid biosynthesis exhibited induced expressions under salt stress (Fig. 4a), suggesting their possible function in controlling ABA synthesis. ABA was a positive regulator to promote the production of liquiritin and glycyrrhizic acid (Seo and Koshiba 2002). In the present study, the ABA content was significantly increased after NaCl stimulation (Fig. 4b), suggesting that there is a close positive correlation between ABA and liquiritin synthesis, and that ABA accumulation and its mediated signal transduction might be a key factor in the regulation of the salt-induced production of secondary metabolites.

Studies reported that salt stress and exogenous ABA treatment could significantly increase the content of endogenous ABA and induce the up-regulation of the expressions of genes related to the ABA signal transduction pathway and promote the synthesis of flavonoids (Zhang et al. 2019; Deluc et al. 2008; Gagné et al. 2011; Enoki et al. 2017). Abscisic Acid-Insensitive 4 (ABI4) as an important factor of ABA signal transduction indicated significant induced expression under salt stress (Luo et al. 2021). ABA was induced in UV-B-treated soybean sprouts, which then resulted in activation of SnRK2 and up-regulation of CHS expression to lead to isoflavone accumulation (Jiao and Gu 2019). *Arabidopsis* and rice SnRK2 kinases can be activated by salt (Boudsocq et al. 2004; Kobayashi et al. 2004). Exogenous application of ABA enhanced PAL activity and increased the accumulation of flavonoids in *Salvia miltiorrhiza* hair roots (Cui et al. 2012). It has been reported the important roles of PAL and CHS for flavonoid synthesis and of CHS and II-CHI for liquiritin synthesis in *G. uralensis* (Yin et al. 2020; Shimada et al. 2003). In our work, the contents of both liquiritin and glycyrrhizic acid appeared significant accumulation in response to exogenous ABA application, and the expressions of the *CHS* and II*-CHI* genes involved in flavonoid biosynthesis, as well as many genes participated in ABA signal transduction were induced (Fig. 5), indicating the important role of ABA in regulating flavonoid accumulation. As far as the genes involved in glycyrrhizic acid biosynthesis were concerned, there were only two genes, *SQS* and *β-AS*, that could be detected with extremely low expression levels, and displayed normal or down-regulated expression after ABA treatment (Fig. 5b), suggesting other regulation mechanisms that control the increase in glycyrrhizic acid, indicating a more comprehensive regulatory network of plant cells.

In conclusion, through the analysis of comparative transcriptomes and metabolites, our results indicate that salt stress could significantly improve the biosynthesis of pharmacological active ingredients in the aerial parts of *G. uralensis*, and that liquiritin accumulation might be regulated via the ABA-mediated signaling pathway. Our study provides candidate key genes and metabolic pathways of pharmacological active ingredients in the aerial part of *G. uralensis* under salt stress, and lays the foundation for further molecular mechanism elucidation of liquiritin biosynthesis and for genetic improvement and comprehensive utilization of *G. uralensis*.

## Supplementary Information

Below is the link to the electronic supplementary material.

Supplementary file1 (DOCX 3833 KB)

Supplementary file2 (XLSX 37 KB)

## Data Availability

All data generated or analyzed during this study are included in this published article and its supplementary information files.
