# Supplementary material for: Integrative analysis of the pharmaceutical active ingredient and transcriptome of the aerial parts of Glycyrrhiza uralensis under salt stress reveals liquiritin accumulation via ABA-mediated signaling
Source: Mol Genet Genomics. 2022 Feb 20;297(2):333–43. doi: 10.1007/s00438-021-01847-1 (PMC8858602; doi:10.1007/s00438-021-01847-1)
Supplement: Supplementary file 1 — Supplementary file1 (DOCX 3833 KB) [file 438_2021_1847_MOESM1_ESM.docx]

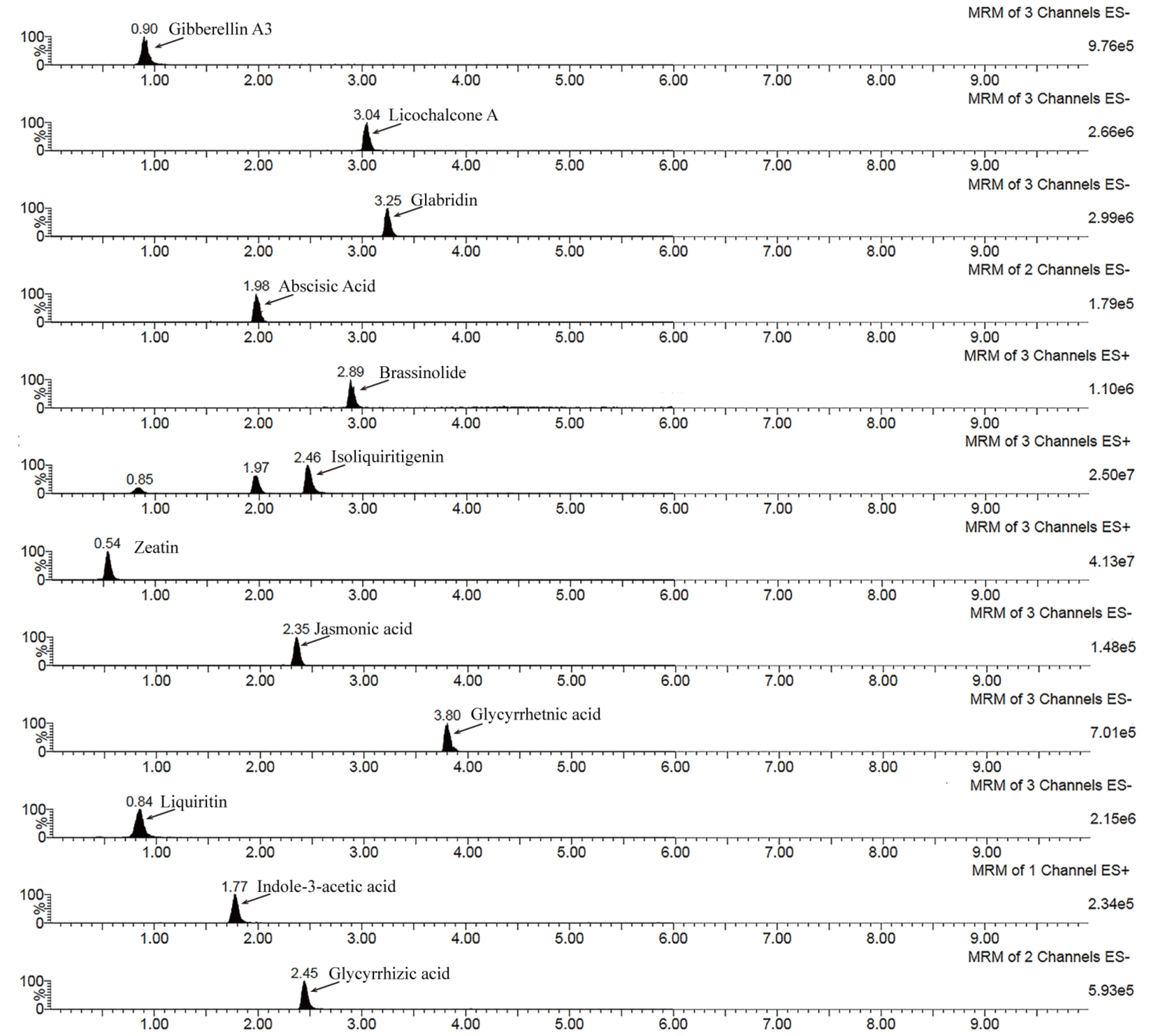


**Supplementary Fig. S1.** Total ion chromatogram (TIC) of the standards of the 12 detected compounds.


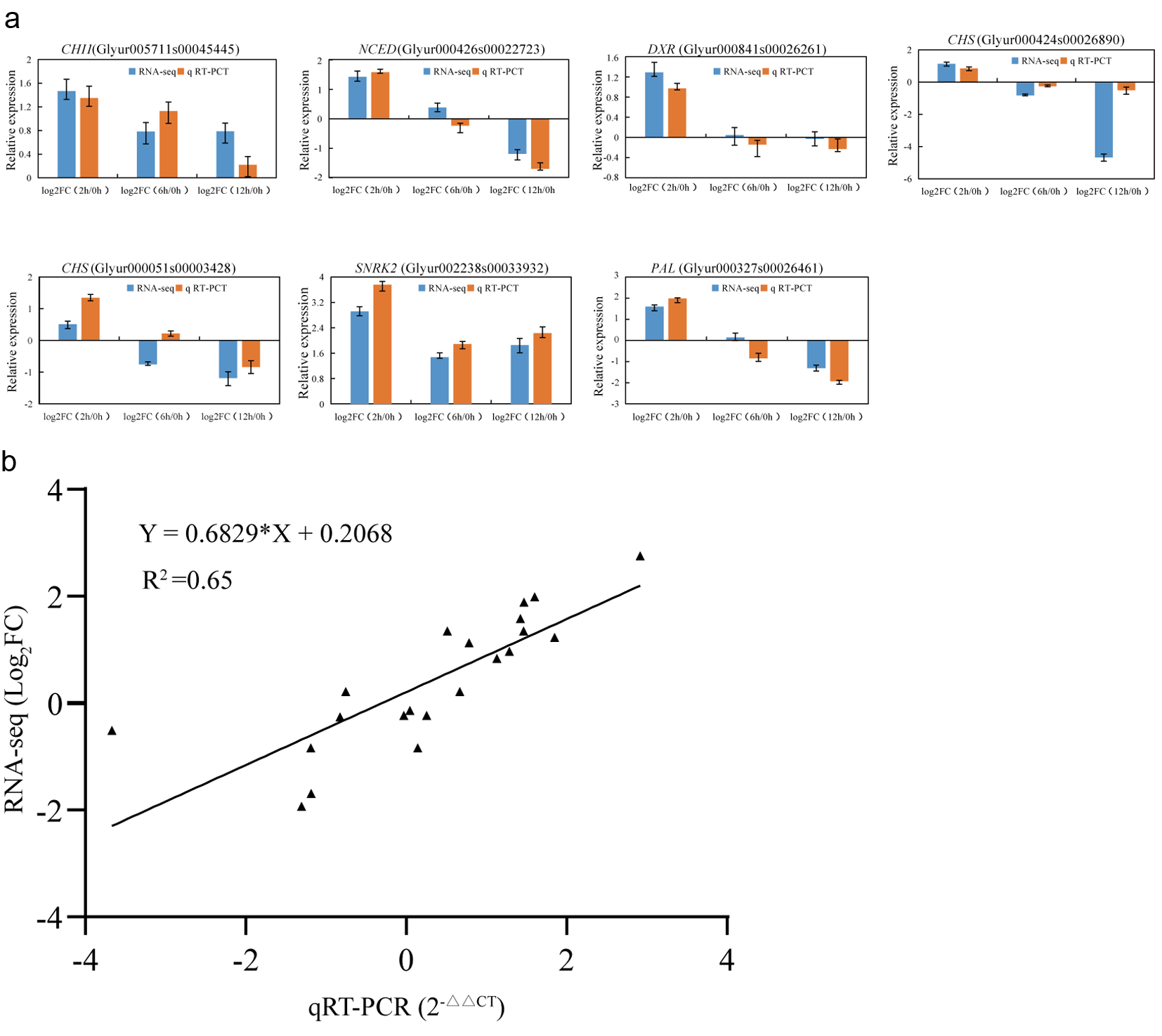


**Supplementary Fig. S2.** Validation of RNA-Seq data by qRT-PCR. a: Comparison analysis of qRT-PCR and RNA-seq using selected genes of *CHI1*, *NCED*, *DXR*, *CHS*, *SNRK2*, and *PAL* that were involved in terpenoid backbone biosynthesis, carotenoid biosynthesis, phenylpropanoid biosynthesis, and plant hormone signaling transduction. b: Correlation analysis of log2 fold change data generated by qRT-PCR with that from RNA-seq.


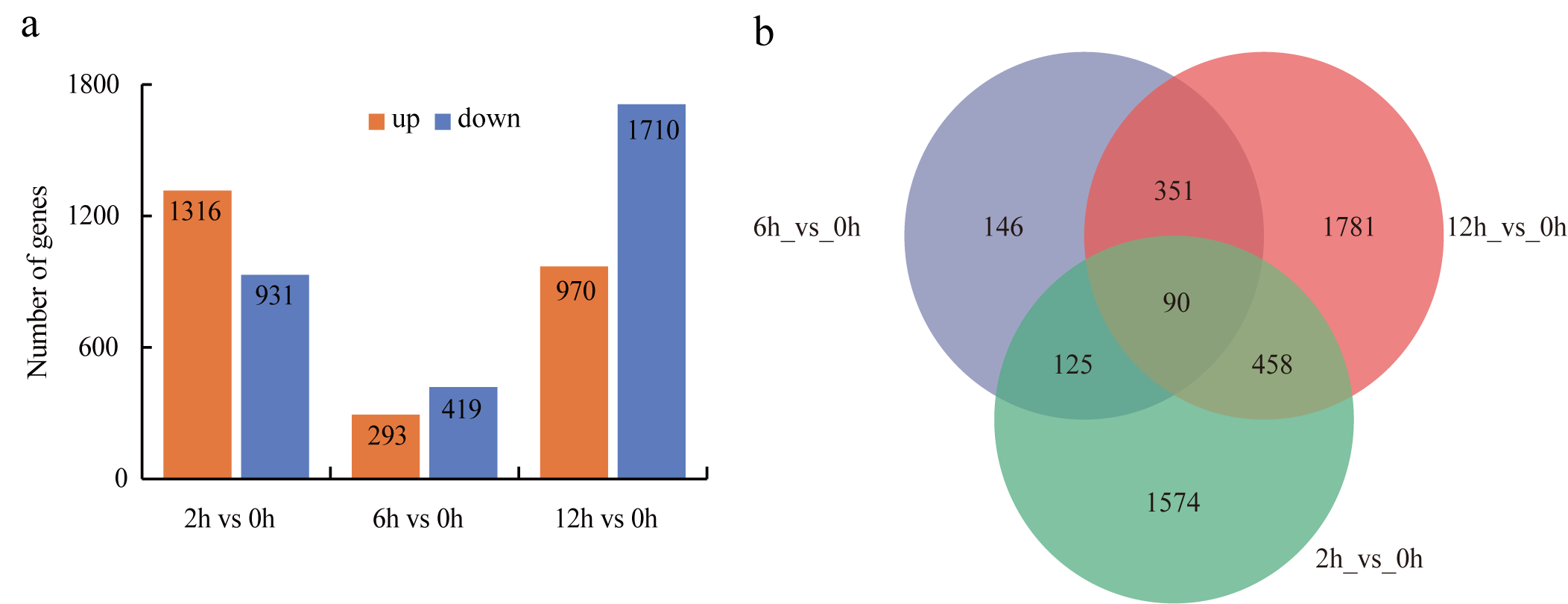


**Supplementary Figure S3.** Statistics and venn diagram analyses of the DEGs in the different comparison groups at different treatment time points by NaCl. a: Statistics analysis of the DEGs identified in different treatment time points under NaCl stress. b: Venn diagram analysis of the DEGs in the three comparison groups at different time of NaCl treatment.


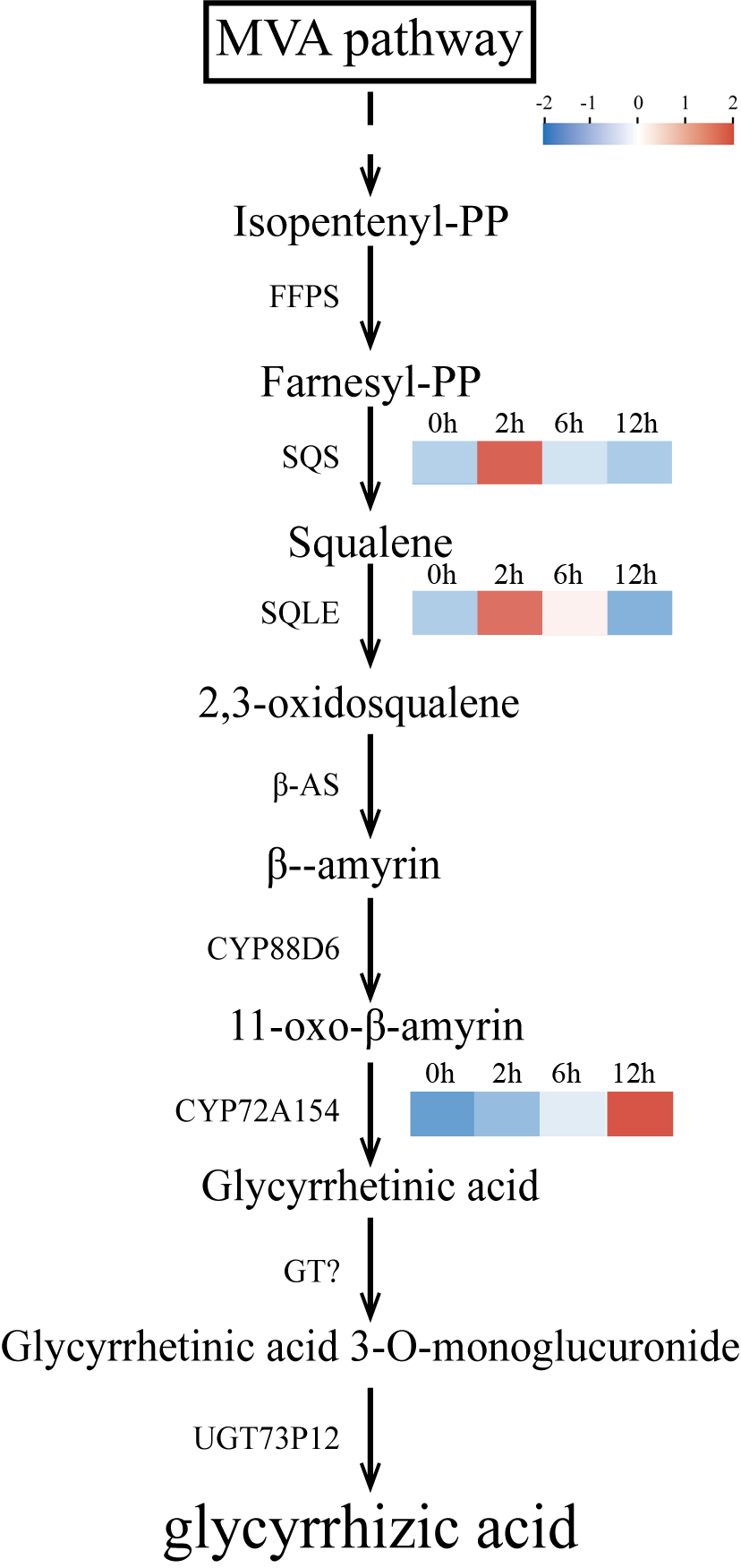


**Supplementary Figure S4.** RNA-Seq-based expression analysis of the DGEs involved in glycyrrhizic acid biosynthesis pathway under salt stress.
